# Diversity, equity, and inclusion considerations for anti-racist, equity-focused NICU family mental health

**DOI:** 10.1038/s41372-025-02493-w

**Published:** 2026-03-16

**Authors:** Emily Echevarria, Tonia Branche, Emily R. Miller, Kimberly Novod, Valencia Walker

**Affiliations:** 1 Department of Pediatrics, Division of Neonatology, Weill Cornell Medicine, New York, NY USA; 2 Division of Neonatology, Ann and Robert H. Lurie Children’s Hospital, Department of Pediatrics, Northwestern University, Feinberg School of Medicine, Chicago, IL USA; 3 Division of Neonatology, Cincinnati Children’s Hospital; Department of Pediatrics, University of Cincinnati, Cincinnati, OH USA; 4 Saul’s Light Foundation, New Orleans, LA USA; 5 Geisinger Commonwealth School of Medicine, Scranton, PA USA

**Keywords:** Paediatrics, Scientific community, Risk factors

## Abstract

Families with infants hospitalized in the neonatal intensive care unit (NICU) experience high rates of perinatal mental health conditions, which are disproportionately experienced by families i) of minoritized racial identities; ii) with a primary language other than English (PLOE); and iii) of low socioeconomic status. Disparities in screening, diagnosis, and treatment for mental health conditions are complex. Multiple individual, interpersonal, institutional, and structural factors may negatively impact marginalized individuals. Multifaceted recommendations to address these challenges are included in this article. This also serves as a call to action for the creation, standardization, and adoption of individual, institutional, and national interventions that can provide equitable mental health support for NICU families to mitigate disparities in mental health outcomes.

## Introduction

An infant’s neonatal intensive care unit (NICU) hospitalization is an exquisitely stressful time for a family and is associated with high rates of depression, anxiety, and post-traumatic stress disorder [1, 2]. For minoritized individuals, there are remarkable disparities observed in NICU infant caregiver mental health outcomes attributable to higher rates of mental health diagnoses and lower rates of treatment [3–9]. Poor caregiver mental health outcomes not only adversely impact affected individuals, they also negatively impact the health and development of their infants [10–12]. To achieve optimal outcomes for NICU patients, in addition to delivering high-quality medical care, the NICU must also prioritize providing high-quality, equitable mental health care to families.

Mental health outcomes are influenced by a family’s cultural background, religious/spiritual beliefs, lived experiences, and ability to access mental health services [3, 9, 13–18]. Healthcare professionals (HCPs) must recognize and consider social, political, and structural drivers of mental health. These drivers encompass a vast array of intersecting personal and social identities, including race, ethnicity, gender, sexuality, physical and cognitive ability, English language proficiency, and socioeconomic status [19]. The focus of this article is disparities in mental health outcomes, specifically related to a NICU hospitalization and interventions to improve them. However, proper attention to mental health disease across the life course, including early preventative mental health care, prenatal mental health care, and post-NICU discharge mental health care, is crucial to improving health outcomes for patients and their families.

As we review care gaps and examine individual, institutional, and national interventions aimed at providing equitable mental health support for NICU families, we incorporate an explicit acknowledgment of the complexity of the factors that contribute to an individual’s mental health. The causes for NICU infant caregiver mental health disparities are multifactorial and incompletely realized. We specifically focus our discussion on those with (i) minoritized racial identity; (ii) primary language other than English (PLOE); and (iii) low socioeconomic status (SES). Many of the other marginalized identities are beyond the scope of this discussion. However, we strongly advocate for attention to them and recommend further research in these areas. We will present several individual, institutional, and state/national practices and policies as early opportunities towards decreasing disparities in mental health outcomes (Table 1).

**Table 1:** Individual, institutional, and state/national recommendations for improving mental health equity in the NICU.

Recommendations for individual clinicians	Examples of action steps
Provide family-centered care that supports families’ involvement, honors their values and beliefs, and incorporates their socioeconomic, racial, ethnic, and cultural background.	• Adapt and modify unit practices to align with families’ work/childcare hours and schedule daily updates, kangaroo care times, therapy visits, and other services accordingly • Create a process whereby families with limited English proficiency can contact the NICU via an interpreter • Adjust staff schedules to ensure ancillary services are also available during evening hours (therapy, social work, lactation)
Employ an equity-focused approach to the development and evaluation of quality improvement initiatives.	• Disaggregate and report process and outcome measures by race and language • Develop interventions to address disparities in processes and outcomes • Co-produce QI interventions with families
Create diverse research teams, reduce barriers to research participation, and provide support to encourage diversity in clinical research.	• Translate research forms into all languages in your patient population • Include interpreter services in your research budget • Create diverse focus groups to assess barriers to research participation
Advocate for universal access to mental health services.	• Work with other neonatal health advocates, such as your state’s children’s hospital association, to submit legislative testimony in support of postpartum Medicaid expansion • Advocate: Call legislators, participate in the AAP advocacy conferences, vote • Subscribe to the AAP’s advocacy updates and alerts: https://www.aap.org/en/advocacy/
Recruit and retain a workforce that reflects the diversity of the patients that they serve.	• Create networks and partnerships with area training programs that include candidates from diverse backgrounds and institutions. • Establish employee equity and support initiatives (i.e., equal pay, diverse representation in leadership, comprehensive benefits) to retain a diverse workforce • Adjust work models and hire staff who have evening and weekend hours
Implement a standardized and universal screening for social and mental health needs and a tiered system for referral and/or intervention within the NICU and to community partners.	• Develop or incorporate an existing social and mental health screening tool for use during admission intake, longitudinally during a hospitalization, and clinic check-ins to capture gaps in patient resources. • Compile a list of virtual and local community partners by resource type for positive screens requiring referral • Hire patient care navigators for the highest risk families to coordinate referrals to necessary services
Evaluate disparities in policies, care structure, patient treatment, and patient outcomes and develop interventions.	• Schedule a quarterly review of data for common care practices (e.g., delivery room skin-to-skin, delayed cord clamping, donor breast milk use) to identify disparities in outcomes for different patient populations (i.e., race/ethnicity, insurance status, language) • Include health equity measures in the review of quality initiatives (e.g., central line infections, pain and sedation medication use, length of stay, etc.)
Develop targeted interventions to eliminate equity gaps.	• Create a standardized protocol with obstetric providers for requesting antenatal NICU consultations to increase the consistency of access to consults for all qualifying families.
Enact federal and state legislation that supports increased access to mental health services and promotes improved mental health outcomes.	• Expand postpartum Medicaid coverage • Require covered benefits for mental health evaluations and treatment by all payer types • Provide paid parental leave

The recommendations in this article were developed as part of the American Academy of Pediatrics (AAP) Trainees and Early Career Neonatologists (TECaN) national advocacy initiative, the Carousel Campaign. The AAP TECaN recognized a lack of standards for screening and treatment of perinatal mental health disorders and organized a multidisciplinary group of neonatal providers and parents of NICU graduates to create best practice guidelines [20]. The topics featured in this article are not comprehensive, but they do illustrate several key issues that contribute to disparities in the mental health outcomes of NICU families. They also serve as a template for recommendations to improve equitable healthcare practice and delivery.

## Background

All families of infants hospitalized in the NICU experience significant amounts of emotional pain and stress and have high rates of depression, anxiety, and PTSD [1, 2]. Mental health outcomes, though, differ by race, primary language, and socioeconomic status [3–9]. The causes for disparities are complex and not fully understood, but likely involve the social drivers of health (SDOH), experiences within the healthcare system (including the NICU), and a person’s life course[21]. An examination of how they contribute to mental health disparities lays the foundation for a discussion of interventions. Recommendations consider improving mental health equity for families as a vital strategy for optimizing child health outcomes [10–12].

There are wide-ranging racialized disparities in postpartum mental health outcomes for birthing people. Non-Hispanic Black women (NHBW) and Latinas are twice as likely to experience postpartum depressive symptoms as compared to non-Hispanic White women (NHWW)[3, 9]. This difference persists even after controlling for a history of depression and socioeconomic factors [3, 4]. Despite higher rates of symptoms, NHBW and Latinas are 40–80% less likely to receive postpartum depression screening, 50% less likely to receive postpartum depression treatment initiation, and over 60% less likely to receive continued postpartum depression care [4, 5]. In qualitative studies of perinatal care, Black women report a need for mental health care and significant difficulties receiving it. Limitations are related to access, advocacy, and distrust [22]. There are few studies on racial disparities in mental health screening and treatment specific to the NICU. Available data suggest that Black, Asian, and Hispanic (Latina) postpartum women are less frequently diagnosed with anxiety and depression as compared to White postpartum women. This occurs despite higher rates of infants admitted to the NICU, a known mental health stressor. With both proven disparities in mental health care access and general utilization, this suggests a gap in care provision for minoritized postpartum individuals rather than a presumption of higher prevalence for mental health diagnoses among White postpartum individuals [23]. A recent study that evaluated mental health-related emergency department visits in birthing people that delivered preterm infants supports this assertion. The study results identified NHB birthing people as almost twice as likely to have a mental health-related visit or hospitalization versus NHW birthing people [24]. A qualitative study of mental health care of Black women with premature infants demonstrated an overwhelming paucity of mental health care and resources, despite a visible and expressed need for support [25]. The limited research available on postpartum mental health symptoms in fathers suggests similar rates of depressive symptoms for NHW, NHB, and Latino fathers [26–28]. Despite this, fathers from minoritized racial/ethnic groups may lag in mental health service use [29], and this area demands more research.

**Fig. 1:**
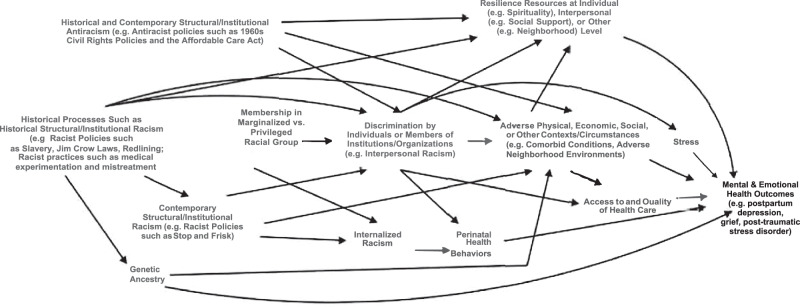
This causal diagram depicts the complex and interrelated factors that influence the relationship between racism and mental and emotional health outcomes. It was adapted from Howe CJ, Bailey ZD, Raifman JR, Jackson JW. *Recommendations for Using Causal Diagrams to Study Racial Health Disparities*. American Journal of Epidemiology. 2022 Nov;191[12]:1981-1989. DOI: 10.1093/aje/kwac140. PMID: 35916384; PMCID: PMC10144617 [33].

Experiences with race-based medical mistreatment, discrimination, abuse, historical experimentation, and exploitation have bred distrust of an untrustworthy healthcare system and contribute to mental health outcomes[30, 31]. Dr. Ruth Wilson Gilmore defines racism as “the state-sanctioned and/or extralegal production and exploitation of group-differentiated vulnerability to premature death [32].” This framework situates experiences with racism as adversely affecting mental and emotional wellness through highly complex interactions (Fig. 1)[33]. Black birthing people report high rates of racism, discrimination, and stress [34]. Black women with infants hospitalized in the NICU, and NICU hospital staff, also report experiencing and observing racism and discrimination [35–37]. Women who report experiences with racial discrimination are 2.7 times more likely to report postpartum depressive symptoms [38]. A history of adverse life events, which minoritized individuals experience at disproportionately higher rates, is associated with the development of postpartum depression [9, 39, 40]. Additionally, women’s experiences with traumatic events, also experienced at disproportionately higher rates by minoritized women, are associated with PTSD symptoms in the perinatal period [41].

Poverty, reliance on public insurance, low educational attainment, unemployment status, living in rural areas, lacking a partner at home, unmarried status, and teenage pregnancy are socioeconomic and sociodemographic factors also associated with increased risk of perinatal depressive and anxiety symptoms [7–9, 42]. Despite an increased risk of symptoms, women of lower socioeconomic status report many barriers to receiving adequate mental health care. Cultural (stigma, language, race, immigration), social (seeking help from social networks, feeling like a burden), physical (lack of childcare or transportation), and systemic barriers (insurance status, financial limitations) result in failure or delay in diagnosis and treatment of mental health disorders [43]. The duration of PPD is dependent on timely diagnosis and treatment, making the consequences of these delays more harmful [44–46].

Although research from the United States is limited, multiple studies show that women with minimal host country language proficiency have higher rates of postpartum depressive symptoms [6, 18, 47]. This is consistent with research on mental health disorders outside the peripartum period, which are also significantly more likely in patients with limited language proficiency [48]. Spanish-speaking parents of NICU patients report barriers to communication that lead to feelings of shame, frustration, and fear [49]. Such experiences can reasonably contribute to higher rates of mental health diagnosis. In a qualitative study of Mandarin-speaking women in Rochester, NY, almost all women experienced peripartum depressive symptoms. Most, however, declined further evaluation or referral for treatment [50]. In Sweden, non-native Swedish speakers were significantly more likely to experience peripartum depressive symptoms, but also less likely to seek treatment [51]. Non-native Portuguese-speaking women’s likelihood of postpartum depressive symptoms increased with decreasing language proficiency[6]. In addition to communication barriers experienced by PLOE patients, qualitative research of both community healthcare workers and PLOE patients demonstrates that care is also compromised by cultural and healthcare access issues [49, 50, 52]. The cultural, language, healthcare access, and social barriers to diagnosis and treatment experienced by PLOE patients lead to miscommunication, feelings of marginalization, and worse health outcomes[49, 50, 52, 53].

Despite the high rates of mental health symptoms experienced by minoritized families, many barriers to diagnosis and treatment remain. The causes for disparate care experiences in the NICU are complex and multifaceted. They can contribute to delays in proper management and poor outcomes. This necessitates a multilevel approach to equitable, anti-racist care to optimize the mental health of NICU families and mitigate disparities.

The remainder of this article will review structure and policy changes to address mental health disparities in NICU families (Table 1). Individual, institutional, and national interventions aimed at improving equity in healthcare provided (standardizing equitable family-centered care, providing universal mental health screening and treatment, ensuring diverse recruitment to research studies, hiring diverse workforces), and also supporting families (providing paid family leave, improving health insurance coverage) can improve disparities in mental health outcomes. Success carries long-lasting benefits for child health and development. Undeniably, no singular recommendation can solve these issues, especially considering the larger inadequacies of the mental health system. Lack of action, however, is not an option. This article focuses on suggested interventions and stresses the importance of partnerships to act with agility in a challenged system.

*A note on mental health diagnoses, evaluation, treatment, and providers:*

No standard exists for delivering equitable mental health care to NICU families. Existing practices vary widely by institution. A prior article in this series, “Understanding and addressing mental health challenges of families admitted to the neonatal intensive care unit” addresses mental health concerns experienced by families and reviews diagnostic tools and interventions utilized by providers [54]. Table 2 also includes a brief overview of mental health diagnoses, screening tools, providers, and resources. Families can have milder experiences with emotional distress or depressive symptoms that do not meet formal diagnostic criteria but nonetheless require evaluation and intervention. A more extensive discussion of diagnoses, evaluation, treatment, and providers falls beyond the scope of this article.

**Table 2:** NICU mental health diagnoses, screening tools, providers, and resources.

Diagnoses and screening tools	Resources	
*Depression* Edinburgh Postnatal Depression Scale Postpartum Depression Screening Scale Beck Depression Inventory Patient Health Questionnaire-9 Patient Health Questionnaire- 2	*NICU- and Hospital-based Providers and Resources* Neonatal physicians, advanced practice providers, nurses Palliative care Psychiatry Psychology Chaplaincy Social work Child life specialists Peer support groups Family advisory committees	*Electronic and Phone Resources* 988 Suicide and Crisis Hotline Hand to Hold Mammha March of Dimes Massachusetts General Hospital’s Center for Women’s Mental Health National Maternal Mental Health Hotline National Perinatal Association National Suicide Prevention Lifeline Postpartum Progress Postpartum Support International Apps: Betterhelp, Moodfit, Sanvello, Talkspace, UCLA Mindful
*Anxiety* General Anxiety Disorder -7 Beck Anxiety Inventory Edinburgh Postnatal Depression Scale - Anxiety		
*Acute Stress Disorder* Stanford Acute Stress Reaction Questionnaire		
*Posttraumatic Stress Disorder* Perinatal PTSD Questionnaire-II City Birth Trauma Scale Impact of Events Scale - Revised Posttraumatic Stress Disorder Checklist		
*Psychosocial Risk (unspecified)* Psychosocial Assessment Tool		

Table adapted from tools and resources suggested by Osborne AD, Yasova Barbeau D, Gladdis T, Hansen K, Branche T, Miller ER, et al. Understanding and addressing mental health challenges of families admitted to the neonatal intensive care unit. Journal of Perinatology [Internet]. 2024;(November). Available from: 10.1038/s41372-024-02187-9 [54].

## Recommendations

### The individual clinician

#### Family centered care (FCC)

*Recommendation: NICU HCPs should deliver universal FCC that supports all families’ involvement in the care of their infant(s), considers their values and beliefs, and incorporates their socioeconomic, racial, ethnic, and cultural identities*.

Family centered care (FCC) is respectful care that honors a family’s values, perspectives, and backgrounds. It incorporates families into care planning and delivery as equal participants, encourages and supports families in caregiving and decision-making processes, and includes transparent sharing of unbiased information with families in their preferred language [55, 56]. FCC in the NICU improves stress, satisfaction, and well-being in families and growth in infants [57–59].

To understand families holistically, HCPs must learn about existing disparities in maternal and neonatal care [60, 61]. HCPs also need awareness of families’ differential quality of care and NICU experience. Minoritized racial groups, families with low SES, and families with PLOE status disproportionately experience gaps in FCC, including inconsistent access to interpreter services, challenges with social work services, increased barriers to presence at the bedside, and difficulties establishing meaningful relationships with nurses [62–66]. NHBW and Latinas report poorer communication and responsiveness from HCPs and less satisfaction with NICU communication overall [64, 65]. Spanish-speaking parents more commonly report misunderstanding aspects of their children’s care and enduring care challenges at discharge related to communication barriers, as compared to English-speaking patients [67, 68]. Consistent use of interpreter and translation services in the family’s preferred language is an important component of ensuring families with PLOE status are involved in the care of their child and understand the care plan, centralizing the team’s efforts on the family’s well-being.

Kangaroo care (KC) is one way to involve families in NICU care with known clinical and mental health benefits for infants and parents [69–75]. Kangaroo care leads to improved vital sign stability, sleep organization, and decreased procedural pain in infants [72, 76, 77]. It also improves bonding, stress, and satisfaction in parents [73, 78]. Unfortunately, its implementation is inequitable: Families of lower SES or PLOE have significantly lower rates, frequency, and duration of KC [79]. Parents from minoritized racial groups report receiving less education about KC as well as less access to it [64].

Breastfeeding and breast milk provision have known health benefits for infants and birthing parents and are important ways to include families in the care of their infant in the NICU [80]. Breastfeeding has been shown to improve mental health, and supporting a family’s breastfeeding goals in the NICU can promote FCC and mental well-being [81]. However, disparities in breastfeeding and breast milk provision pose a threat to the mental health of minoritized birthing parents and families who are already at high risk of inequitable support and discrimination [64, 82–86]. Minoritized birthing people are at increased risk for lower rates of breastfeeding initiation and higher rates of breastfeeding cessation [80, 83, 86–88] for multiple and interrelated reasons. These include historic injustices [89], inadequate access to institutions and services that support breastfeeding and breastmilk provision [90], and a dearth of policies that support equitable breast milk provision, like paid parental leave (PPL) and breastfeeding breaks [91]. Thus, intentional and equitable breastfeeding support may facilitate efforts to decrease disparities in mental health outcomes. More research is needed for an adequate understanding of the complex and bidirectional relationship between breastfeeding and maternal mental health outcomes [92, 93]. Table 3 includes key factors and offers research questions regarding the unique relationship between breastfeeding, mental health, and social inequity. Admittedly, the more detailed discussion of this multifaceted interaction expands beyond the scope of this manuscript and deserves dedicated exploration.

**Table 3:** Considerations regarding the relationship between mental health disparities and breastfeeding in the NICU [81, 90, 91, 189–191].

Key factors	Suggested research question
Depressive and anxiety symptoms linked to lower breastfeeding initiation and early cessation	How do prenatal and postpartum mental health conditions impact breastfeeding initiation and duration?
Early initiation and longer breastfeeding duration associated with improved mental health outcomes	Can interventions that support breastfeeding improve maternal mental health outcomes in populations intending to breastfeed?
Inability to breastfeed NICU infants leads to parental feelings of loss and grief	What mental health interventions are most effective for NICU parents experiencing breastfeeding challenges?
Racial disparities in breastfeeding rates (e.g., lower rates among NHB individuals compared to NHW and Latina)	What structural factors contribute to racial disparities in breastfeeding rates and outcomes?
Lower breastfeeding rates among individuals with low SES	How do socioeconomic factors influence access to breastfeeding resources and support systems?
Inadequate institutional and service support for breastfeeding	How does access to breastfeeding-friendly healthcare and community services affect breastfeeding duration and success?
Lack of equitable policies that support breastfeeding and breast milk provision (e.g., paid parental leave, lactation breaks in the workplace, insurance coverage and provision of effective breast pumps)	How do family support policies (e.g., paid parental leave) impact breastfeeding equity across racial and socioeconomic lines?

Despite the well-documented benefits of FCC components such as breastfeeding and KC, best practice guidelines for delivering FCC in the NICU are not well established. In 2017, Davidson and colleagues organized a multidisciplinary team, including patients and families, to use existing evidence and previously published patient-centered clinical practice guidelines to create FCC guidelines for neonatal, pediatric and adult intensive care units [94]. The California Perinatal Quality Care Collaborative (CPQCC) developed suggestions for promoting FCC for diverse families in the NICU in key areas including “acculturation of the unit; staff communication; counseling; organizational resources; family leadership; and education” [95]. Based on this literature, Table 4 outlines NICU FCC principles to implement for increasing familial involvement in infant care, expanding staff education and training, and providing resources from an institutional level [56, 94–96]. Continued research in this area and involvement of NICU parents and families in development of FCC guidelines can help further establish best practices for FCC in the NICU. Quality improvement initiatives and multidisciplinary collaboratives that include family members can help advance FCC guidelines and programs, ensuring the family voice is meaningfully represented in the development process. NICUs should track metrics to assess the performance of their FCC initiatives and disaggregate outputs by sociodemographic and socioeconomic variables to determine optimal outcomes across all demographic groups.

**Table 4:** Operationalization of equitable FCC in the NICU at the family, staff, and organization level [56, 94–96].

Key elements of FCC	Family	Staff	Organization
Family-centered visitation, rounds, and care meetings	Conduct rounds at patients' bedsides or in a designated room equipped to include families' presence Provide opportunities for siblings and other family members to visit or connect remotely during patients' stays Conduct rounds in families' preferred language with interpreter services	Provide formal staff communication training so they may effectively involve families in interdisciplinary rounds and meetings Incorporate interpreters at a regularly scheduled time for rounds and in-person for meetings	Adopt flexible visitation policies that allow families to be present for rounds or meetings based on their preferences and schedules Utilize virtual technology for families to join rounds remotely Establish contracts with in-person interpreters in various languages
Family-centered developmental care	Involve families in bedside care and parenting tasks (e.g., reading, KC, feeding, diaper changing, physical/occupational therapy)	Educate staff on eliciting families' participation in bedside care of infants and the importance of early relational health Create educational sessions and activities for families related to infant care	Develop guidelines and protocols for safe involvement of families in the care of infants with complex medical needs
Supporting use of breast milk	Connect families to inpatient and outpatient lactation support services Establish families' feeding preferences on admission and reevaluate throughout hospitalization	Train staff as lactation consultants to improve access of families to lactation support Educate staff on benefits of breast milk and breastfeeding	Provide hospital-grade breast pumps in every patient bedspace Create donor milk program to serve as bridge to maternal breast milk
Mental health support	Create peer-to-peer support groups at various times of day and solicit families' participation Connect families to mental health resources as indicated by mental health screening	Use validated tools to train staff on communication skills for conducting difficult news and goals of care conversations Perform mental health screening at regular intervals throughout stay	Provide adequate sleep environments or housing options for families to be near infants and well-rested Hire an adequate number of mental health providers to cover the NICU
Cultural humility	Elicit families' cultural preferences regarding infants' care and stay (e.g., preferred communication method and timing, spiritual/cultural practices, clothing and body care materials, etc.)	Require staff cultural competency training with onboarding and maintenance of certification Develop educational materials in multiple languages and culturally relevant formats	Utilize inclusive signage and education materials Hire diverse staff reflective of the unit demographic
Social support	Connect families to social resources as indicated by social screening results Include families' support systems for critical moments in care Provide referral to and/or establish partnership with doulas and other postpartum support personnel	Perform social determinants of health screening at regular intervals throughout stay Collaborate with family support personnel (i.e. doulas)	Hire an adequate number of social workers and family care navigators
Family Advisory Councils (FAC)	Solicit and include families' perspectives in development of unit initiatives, research, and guidelines Provide referral to and/or establish partnership with doulas and other postpartum support personnel	Include FAC when initiating new guidelines and programs	Establish diverse FACs with appropriate compensation and training

#### Quality improvement and research

*Recommendation: Equity-Focused Quality Improvement (EF-QI) projects should be used to improve FCC and mental health disparities in the NICU. Investigators must create research teams that reflect the racial/ethnic communities served, reduce barriers to research participation by patients from minoritized communities, and provide support to encourage increased participation of racialized minorities in clinical research*.

EF-QI initiatives can increase the provision of equitable care [61, 97, 98] and target disparities in mental health outcomes within the NICU. Recent QI studies show that QI methodology effectively improved the delivery of FCC in the NICU [10] and decreased disparities in breast milk provision [99]. EF-QI can also be used to improve mental health screening and intervention in the NICU. Overall improvements in care quality often decrease disparities [100, 101]. Disaggregation of outcomes data by race, language, or SES, among other factors, is necessary to ensure interventions do not inadvertently exacerbate disparities. Additionally, inclusion of families and community stakeholders in the QI team can increase awareness of local inequities and barriers to improvement, thus optimizing disparity reduction. This is outlined in the EF-QI framework proposed by Reichman et al. Their framework intentionally integrates concepts of health equity into every aspect of the QI process to avoid intervention-generated inequalities that further harm groups already made vulnerable by racism and other forms of marginalization[98].

When conducting NICU-based clinical research, researchers should intentionally design studies to ensure representation from minoritized populations. Additionally, they should create study cohorts that represent existing NICU populations, which are skewed toward NHB patients [102–104]. The resulting research findings are then more likely to be applicable and generalizable. Reasons for lower rates of participation in research studies by minoritized populations include knowledge of past abuses by the research community leading to mistrust, less access to healthcare centers where studies are conducted, lower likelihood of being asked to participate in research studies, exclusion criteria that limit participation, and fear of discrimination [105–107]. Researchers must (1) assess barriers and unique characteristics that individuals from different backgrounds experience and possess that limit their research participation (PLOE, lack of transportation or access to technology, challenges with health literacy, disabilities, distrust of the scientific community), and (2) provide targeted support and outreach to overcome these barriers and accommodate their specific needs. Recommendations for equitable and inclusive NICU research practices are listed in Table 5. Importantly, it is not solely the responsibility of individual researchers or research teams to ensure representation of minoritized racial/ethnic groups in research. Systemic changes through policy, community, and institutional initiatives are needed [108]. This may include required training on equitable research practices, education on the history of exploitation of minoritized communities for research purposes, non-negotiable inclusion of community advisory boards when developing and implementing research protocols, and use of focus groups to assess acceptability of research methods within diverse communities.

**Table 5:** Recommendations for equitable and inclusive NICU research practices [102–108].

Research project elements	Current practices	Recommended expanded practices
Study population	Efforts to recruit diverse NICU populations; general awareness of disparities in participation	Intentionally design studies with representative cohorts reflecting the NICU population, especially NHB infants
Study recruitment	Targeted recruitment strategies used in specific studies	Develop and implement targeted support and outreach strategies to accommodate the specific needs of underrepresented communities
Inclusive study design	Examination of general bias toward certain outcomes in research analysis methods	Use of study tools that are designed with reduced measurement bias against certain populations and appropriate health literacy level
Barriers to participation	Limited recognition and inconsistent assessment of specific barriers faced by minoritized populations	Assess and address barriers unique to different groups (e.g., PLOE, transportation needs, access to technology, low health literacy, disabilities, and distrust in medical system)
Responsibility and accountability	Responsibility for inclusive research practices often placed on individual researchers or teams	Broaden responsibility to include institutional, community, and policy-level initiatives for inclusive research practices
Research team training and education	Training on human subjects and IRB processes Limited focus on equity and historical context	Mandate training on equitable research practices and the history of exploitation of minoritized communities
Community engagement	Occasional community involvement in study design or dissemination Participant advisory board created after research study has begun	Require inclusion of community advisory boards and conduct focus groups at study inception to ensure research methods are culturally acceptable and trusted Include translation of knowledge back to the community as part of research process

Many questions remain unanswered regarding disparities in NICU family mental health that demand further research. Prospective, population-based studies must be done to further examine drivers for disparities in NICU family mental health outcomes and equitable interventions to improve them. More research is needed on the effects of mental health challenges that may not meet formal psychiatric diagnostic criteria yet still challenge the well-being of NICU families. Such examples include experiences of disempowerment, estrangement, and discrimination. Developing more validated tools, like the Family Cultural Wealth Survey, is important to quantify families’ sources of resilience and support systems. These can be adapted to the NICU setting and reinforced by providers and the community to position families for greater success during their NICU experience [109]. Culturally sensitive methods to support families with unique sociocultural mental health needs based on their lived experience should be developed and evaluated. Additionally, the mental health needs of other marginalized communities, including fathers and non-birthing parents, adoptive parents, LGBTQIA+ parents, parents living with disabilities, and non-traditional parents (grandparents, etc.) demand further research. A more comprehensive characterization of the drivers perpetuating mental health disparities among diverse NICU families will serve as a foundation for interventions and policies designed to mitigate them.

### The institution

#### Workforce

*Recommendation: Hospitals should employ a workforce that reflects the diverse identities of the patients they serve, including gender, race, ethnicity, language, religion, age, sexual orientation, physical abilities, and ideologies*.

Despite calls by the Institute of Medicine in 2003 and 2024 to improve workforce diversity to combat health inequities[110, 111], Black, Hispanic, and Native American individuals remain underrepresented across healthcare professions [112]. Racial and language concordance literature shows improved patient experience, patient-provider communication, and patient outcomes across broad domains and warrants additional investigation as a strategy for improving persistent health disparities[110, 111, 113].

Potential drivers for racial concordance improving patient outcomes may include increased satisfaction with care [114, 115], enhanced trust [116], favorable perception of shared experience/understanding[117], and better adherence to treatment recommendations [118]. Black patients with racially concordant providers are more likely to receive postpartum care [119]. This is a critical timepoint for assessing postpartum maternal mental health. Research indicates that Black women prefer racially concordant perinatal and mental health providers. They report building closer relationships and making greater progress in their mental health treatment [34, 120]. Black women with infants hospitalized in the NICU expressed the importance of having providers that resemble them [25, 37]. Ethnic concordance of Latin patients during mental health visits led to higher continuance of care (attending next session) and higher working alliance as compared to discordant dyads [121, 122]. Latin individuals were also more likely to seek preventive care, care for a new problem, and care for an ongoing problem when seen by ethnically concordant providers [123].

In addition to the potential for benefits conferred with racial-concordant care, language-concordant care offers an important method for physicians to meet the unique needs of patients and families with PLOE status [124]. Despite the importance of communicating with PLOE patients in their preferred language, Spanish-speaking families in the NICU report that medical teams failed to communicate with them in their primary language during the majority of interactions [68, 124, 125]. After delivery, women with the desire to breastfeed are more likely to leave the hospital exclusively breastfeeding when cared for by language-concordant nurses [126]. Spanish-speaking mental health patients strongly preferred bilingual providers as compared to using an interpreter, citing improved trust, communication, and privacy [127]. Likewise, bilingual mental health providers felt communication improved when there was language concordance with their patients[127]. A study of Asian Americans with limited English proficiency determined that language discordance with their provider led to lower likelihood for patients to ask questions they wanted to ask about their mental health [128].

On an individual level, language and racial concordance may improve patient uptake of mental health care, patient experience, and mental health outcomes. Racial and language concordance between providers and patients may also diminish cultural and language barriers to care described by minoritized communities. On a larger scale, greater diversification of the workforce may decrease medical distrust [129], mitigate racist experiences [130], and improve health equity [110, 111]. Simply ascribing to a workforce diversity quota will not yield benefit. All HCPs must work to understand individuals’ cultural backgrounds, unique lived experiences and perspectives, and communication styles and adjust their care accordingly [22, 114, 130]. Incorporating evidence-based, culturally sensitive, antiracist tenets into health professions education and continuing education for the healthcare workforce is essential [22].

#### Health equity education and other institutional interventions

*Recommendation: Hospitals should evaluate explicit and implicit disparities in their policies, care structure, patient treatment protocols, and patient outcomes and develop targeted interventions to eliminate health equity gaps*.

Research demonstrates significant disparities in healthcare quality and outcomes both between and within hospitals [131]. Hospitals should evaluate the disparities that exist in their patients’ mental health care quality, experiences, and outcomes to develop effective and targeted interventions [132, 133]. Patient, quality, and outcome data require disaggregation to uncover disparities. Interventions should be specific, intentional, and problem-oriented. They may include standardizing an equity-focused framework into research projects and QI initiatives [134], amending hospital care structure and policies to deliver more equitable care [135], and/or employing local families or community engagement specialists to illuminate previously unrecognized systemic barriers to achieving optimal outcomes [136, 137]. Although this requires a substantive funding commitment, cost effective analyses can demonstrate the value and savings of a robust care system that optimizes outcomes for all patients through strategic resource allocation structured for individualized patient needs.

Hospitals may join perinatal quality collaboratives (PQCs): The goal of these state-based collectives of stakeholders is to identify and improve caregiver and infant health outcomes by reducing disparities through QI [138]. PQCs provide an infrastructure for hospitals to create and implement QI projects [139]. PQC initiatives have reduced disparities in post-partum hemorrhage[140] and are currently directed at improving caregiver mental health outcomes [138]. The Health Resources and Services Administration (HRSA) and ACOG have partnered to create the Alliance for Innovation on Maternal Health (AIM). Their QI initiative offers freely accessible care bundles targeting perinatal mental health conditions. Health equity is integral to its foundation. Hospitals not actively participating in this initiative can use AIM resources and apply for funding to achieve equitable maternal mental health outcomes [141].

In recent decades, hospitals and medical schools have incorporated implicit bias and cultural competency training with the goal of achieving equitable patient outcomes. While current research suggests these strategies may not alter patient outcomes, they may enhance cultural awareness and improve other aspects of patient care experiences (e.g., communication and satisfaction)[142, 143]. In racial concordance studies and qualitative studies of Black patients’ experiences in the NICU, Black patients emphasize the importance of providers that display cultural humility and awareness of racial inequality, especially when racial concordance of providers is not possible [25, 120]. Institutions seeking implicit bias training for employee education can access free online resources [144, 145]. The shift towards structural competency training adds emphasis to social and structural forces that drive patient outcomes and disparities beyond the individual level [146]. To foster cultural competency, hospitals can collaborate with external sectors and stakeholders, partner with community-based organizations, and create innovative health promotion strategies. The MedEd Portal provides a freely accessible cultural competency curriculum for healthcare teams to learn about cultural competency and develop strategies for interventions that can work both within and outside of the healthcare settings [147].

#### Universal screening and referral: social and mental health needs

*Recommendation: Hospitals should standardize and implement universal screening for social and mental health needs and utilize a tiered system for referral and/or intervention, within the NICU and out to community partners*.

##### Social drivers of health screening

The SDOH impact both disparities in neonatal outcomes [148] and disparities in NICU family mental health outcomes. Experiences with racism, lower educational attainment, lower SES, PLOE, and rural location are frequently recognized risk factors for stress, anxiety, and depressive symptoms after an infant is born. They are also associated with increased barriers to care [6, 7, 9, 18, 38, 47, 149]. Mounting evidence correlates SDOH screening with increased receipt of needed resources and improved patient outcomes [150, 151]. The AAP recommends SDOH screening in clinical settings [152, 153], and NICU HCPs believe screening is both necessary and feasible [154]. However, SDOH screening remains largely underutilized in the NICU setting [155].

No screening tool for identifying and managing unmet needs has been specifically developed and standardized for use in NICUs. To address this gap, NICU HCPs have adapted publicly available screening tools for NICU use [154–157]. By identifying patients’ social needs, screeners enable NICU HCPs to offer more comprehensive and personalized care. However, they also present potential drawbacks: potential for stigmatization, exacerbation of time constraints, and logistical challenges to implementation, including timing of screening and follow-up. Creative solutions may need to be developed to overcome some of these challenges. Providers may create multidisciplinary teams to develop institution-wide protocols. These teams may include nurse practitioners, physician assistants, psychologists, psychiatrists, nurses, social workers, unit clerks, and community partners. Team members can perform screening, provide resources to patients, and follow up with patients. Based on a recent systematic review of SDOH screening tools [158], web-based instruments can help interested stakeholders compare and select social risk assessment tools most appropriate to their setting and population [159, 160]. Table 6 provides a brief summary of common SDOH screening tools.

**Table 6:** Social determinants of health screening tools [152, 159, 160].

	Education access and quality	Health care and quality	Neighborhood and built environment	Social and community context	Economic stability	Food	Notes
iHELP	x		x	x	x	x	7th grade reading level https://sirenetwork.ucsf.edu/sites/default/files/IHELP_QUESTIONS.docx
PRAPARE	x	x	x	x	x		8th grade reading level Available in 25 languages Designed for adult population https://prapare.org/
SEEK			x	x		x	4th grade reading level Available in 7 languages https://seekwellbeing.org/
SWYC	x		x			x	10th grade reading level Available in 19 languages https://www.teamupforchildren.org/swyc/
WE CARE	x		x		x	x	9th grade reading level Available in English and Spanish https://www.bmc.org/pediatrics-primary-care/we-care/we-care-model

##### Mental health screening

Parents of infants hospitalized in the NICU experience high rates of mood disorder symptoms, with over 40% of mothers and fathers experiencing postpartum depression and anxiety symptoms [1, 161]. The ACOG [162] and the AAP [163] recommend universal mental health screening (Table 2). Early and repeated universal screening is critical to providing needed care. The NICU can be an ideal location for this to occur as postpartum patients with an infant hospitalized in the NICU make many more visits there than to their own providers, such as their obstetrician or primary care physician. No risk factors can accurately predict postpartum psychiatric illness [161, 164, 165], but women asked about their mental health in the postpartum period are significantly more likely to receive mental health counseling [166]. Almost all states now recommend or require screening for postpartum depression [167, 168], though challenges remain. One major concern is the lack of standards, such as choice of test to use, timing, frequency, administration to both parents, and recommendations for management of positive results [161, 164–166]. More research is also needed to standardize evaluation and treatment of mental health disorders in non-birthing parents and others providing infant care.

In considering the prevalence of psychiatric symptoms in NICU parents, it is reasonable to conclude that screening alone is inadequate. There are published standards for social work, psychology, and psychiatry support in the NICU, and recommendations regarding parent education and peer support groups [169, 170]. Universal interventions, however, are likely needed to adequately support the mental health needs of NICU families. We acknowledge that not all care settings can provide this level of support. Approaches to screening and intervention can still be adapted to optimize local resources, including the creation of parent support groups, collaboration with community partners, and the utilization of other providers and staff to take part in screening, referral, and follow-up (as described in the SDOH section). Telemedicine can also increase accessibility to evaluation and treatment in lower resource settings [171–173]. Use of AI is another area that warrants attention [174, 175].

## State and national

*Note: The authors acknowledge the myriad policies that contribute to mental health challenges in the United States. It is beyond the scope of this paper to dissect how exposure to unfavorable social, economic, geopolitical, and environmental circumstances—including poverty, violence, inequality, and environmental deprivation—increases people’s risk of experiencing mental health conditions and exacerbates disparities in mental health outcomes*[176]*. Opportunities to improve prevention, diagnosis, and treatment of perinatal mental health conditions include a number of social and economic policies that increase access to and reimbursement for services, enhance data and accountability, and support families. We have chosen to focus on two evidence-based policies which can reduce longstanding disparities in access to mental health services—optimal insurance coverage and access to supportive family policies*.

### Health insurance

*Recommendation: Extend Medicaid coverage in the postpartum period and increase payer reimbursement for mental health services, including community- and telehealth-based services*.

In a cross-sectional analysis of insurance status at delivery, Daw et al found wide variations in payer type by race and ethnicity. Medicaid was the source of payment for 67% of Latina and 66% of NHB births, compared to 30% of NHW births. Conversely, commercial insurance covered only 19% of Latina and 32% of NHB births, compared to 68% of NHW births [177]. By current convention, medical insurance is necessary and essential for postpartum individuals to receive screening, diagnosis, and treatment for mental health and other chronic conditions.

Historically, Medicaid maternity coverage ended at sixty days postpartum, diminishing enrollees’ access to mental health services compared to those with commercial insurance [178]. In addition, adequate Medicaid and commercial reimbursement for varied care models, including community- and telehealth-based services, is critical for reducing barriers to care. These payer differences reinforce health inequities that contribute to disparities in postpartum psychiatric illness diagnosis and treatment [179]. As of May 2025, at least 49 states have extended postpartum Medicaid coverage to twelve months, and 41 states have adopted expanded income eligibility [180]. These recommendations cannot exist devoid of existing infrastructure deficits. They can, however, serve as an impetus to address limitations in receiving mental health care due to insurance transitions after delivery, critical HCP shortages, and fragmented prenatal and postpartum care.

### Family support

*Recommendation: Promote state and national paid family and medical leave policies and access to high-quality, affordable childcare, which support the health and well-being of infants admitted to the NICU and their parents*.

Many health disparities are deeply rooted in racialized practices and policies that manifest throughout the public sector, including access to quality healthcare, housing, employment opportunity, education, transportation, and policing. Related to employment and childcare, PPL and access to affordable childcare are strongly linked for families with infants in the NICU.

The United States is one of the few developed countries that does not offer PPL. As of May 2025, only ten states have fully implemented a paid family and medical leave program of at least twelve weeks for parents who give birth and at least six weeks for all other parents with a new child [181]. Without access to employer-sponsored or state-mandated PPL, people rely on the Family and Medical Leave Act (FMLA), which provides unpaid, job-protected leave. In 2024, more than 7 million people (two-thirds of eligible applicants) did not take needed leave because they could not afford it [182].

Multiple research studies demonstrate that PPL is associated with improved mental health outcomes in mothers [183].In one study, researchers found that >12 weeks of PPL was associated with a 15% reduction in postpartum depression [184]. Possible mechanisms include increased NICU visitation, parental bonding, and skin-to-skin contact [185]. Access to affordable childcare can similarly overcome time- and resource-related barriers to NICU visitation, thus increasing parental presence and improving mental health outcomes [186].

However, there are significant inequities by gender, race and ethnicity, and family structure. Women, workers of color, and solo parents are less likely to take needed leave [187] or afford high-quality childcare [188]. This may contribute to mental health inequities and provide a partial explanation for the previously described higher risk for postpartum depressive symptoms identified in previous research studies [7–9].

## Conclusion

Parents and caregivers with an infant in the NICU experience high rates of stress, anxiety, and depressive symptoms. These challenges are often intensified by gaps in communication and inconsistencies in care delivery. Families of minoritized racial and ethnic backgrounds, PLOE, and low socioeconomic status disproportionately endure these difficulties as they navigate racial, ethnic, and cultural discordance within the NICU environment. To further complicate concerns about disparate mental health outcomes, evidence suggests that manifestations of racism, both beyond and within the NICU, may act as drivers for inequities, regardless of whether this is acknowledged or understood by those without similar lived experiences.

Significant variability in screening and treatment of mental health disorders can impact whether NICU families receive care, how often they engage in services, and the quality of the care they receive. This variability is often related to a family’s racial, language, and socioeconomic background, which can trigger unequal pathways that promote or hinder a family’s mental health care. These inequities accumulate  and result in stark disparities in mental health outcomes of NICU families.

Equity-focused, anti-racist, and evidence-based interventions are essential for eliminating mental health disparities related to race, ethnicity, socioeconomic status, and preferred language. These strategies can be applied at individual, institutional, and national levels and are critical for optimizing the health and well-being of NICU families and their infants.

### Nomenclature

Throughout this article we use the term “caregiver” and “parent” to describe the primary caregiver(s) and loved ones of the infant in the NICU. When the term “mother” or “father” is used, it describes the terminology as used by the study referenced. We realize that a more inclusive concept of families accounts for a variety of relationships and connections that may fall outside of a “traditional” family unit. We make efforts to be sensitive to and respectful of occurrences where nomenclature used may not describe all types of families. Similarly, we acknowledge the debates and disagreements about defining racism. We structure this discussion by accepting the existence of overt and insidious manifestations of racism in U.S. society. We stratify racism as internalized, interpersonal, institutional, and structural to categorize how it exacerbates the challenges and complexities of a scholarly approach to this topic. We refute the application of race as a biological construct and more aptly apply it as a social construct that can act as a proxy for racism. We further frame experiences with racism as key contributors to social drivers of health, health inequities, and disproportionately adverse health outcomes. We propose that adopting anti-racist, equity-focused actions to interventions aimed at improving health outcomes in the NICU are integral to success, particularly where outcomes remain disparate for minoritized and mistreated populations.
